# CO_2_-Extract-Based Phytopreparation from *Arctium tomentosum* Mill. Root Exhibits No Acute and Subacute Toxicity and Suppresses Xylene-Induced Ear Edema and LPS-Induced Acute Inflammation in Mice

**DOI:** 10.3390/molecules31111900

**Published:** 2026-06-01

**Authors:** Arailym Aitynova, Moldyr Dyusebaeva, Diana Issayeva, Gulzat Berganayeva, Nailya Ibragimova, Alya Berganayeva, Tamara Shalakhmetova, Bogdan Sevastre

**Affiliations:** 1 Faculty of Biology and Biotechnology, Al-Farabi Kazakh National University, 71 Al-Farabi Ave., Almaty 050040, Kazakhstan; arailym.aitynova@gmail.com (A.A.); tamara.shalakhmetova@kaznu.kz (T.S.); 2 Faculty of Chemistry and Chemical Technology, Al-Farabi Kazakh National University, 71 Al-Farabi Ave., Almaty 050040, Kazakhstan; gulzat-bakyt@mail.ru (G.B.); aberganayeva@bk.ru (A.B.); 3 Scientific Center for Anti-Infectious Drugs, 75B Al-Farabi Ave., Almaty 050060, Kazakhstan; diana.isaeva.99@inbox.ru; 4 Faculty of Engineering and Information Technologies, Kazakh-German University (DKU), Almaty 050010, Kazakhstan; 5 Department of Clinic and Paraclinic Sciences, University of Agricultural Sciences and Veterinary Medicine, 400372 Cluj-Napoca, Romania; bogdan.sevastre@usamvcluj.ro

**Keywords:** CO_2_ extract, *Arctium tomentosum* Mill., acute toxicity, subacute toxicity, xylene, ear edema, lipopolysaccharide, inflammation, anti-inflammatory effect

## Abstract

A phytopreparation based on the CO_2_ extract of *Arctium tomentosum* Mill. root (AT) was evaluated for its safety and anti-inflammatory potential in Swiss albino mice. Acute and 28-day subacute oral toxicity studies demonstrated that AT, at doses up to 5000 mg/kg (acute) and 400 mg/kg (subacute), did not induce mortality, clinical signs of toxicity, or adverse effects on body weight, relative organ weights, or hematological and biochemical parameters. Histopathological analyses confirmed preserved tissue architecture in major organs, indicating the absence of structural toxicity. Anti-inflammatory activity was assessed using xylene-induced ear edema and LPS-induced systemic inflammation models. AT significantly reduced ear edema and suppressed the production of pro-inflammatory cytokines (TNF-α, IL-6, and IL-1β) in a dose-dependent manner. Additionally, AT exhibited potent hepatoprotective and nephroprotective effects, as reflected by the stabilization of ALT, AST, SCr, and BUN levels. Histological examination of inflamed tissues corroborated these findings. Overall, AT is well tolerated and demonstrates potent systemic anti-inflammatory and multi-organ protective properties, supporting its potential as a promising therapeutic candidate for inflammatory and oxidative stress-related conditions.

## 1. Introduction

Medicinal plants remain an important source of bioactive compounds with therapeutic potential, particularly for the management of inflammatory disorders [1,2]. Chronic and acute inflammation underlies the pathogenesis of numerous diseases, and despite the widespread use of non-steroidal anti-inflammatory drugs (NSAIDs), their long-term application is often limited by adverse effects, including gastrointestinal, hepatic, and renal toxicity [3]. This has stimulated growing interest in plant-derived preparations with favorable safety profiles and multi-target mechanisms of action [4].

*Arctium* (Asteraceae) is a genus of medicinal plants traditionally used for the treatment of inflammatory conditions, skin disorders, and metabolic disturbances [5]. Roots of *Arctium* species are rich in polyphenolic compounds, flavonoids, and other secondary metabolites known to modulate inflammatory signaling pathways and oxidative stress [6]. While *Arctium lappa* L. is the most widely recognized and studied member of the genus, *Arctium tomentosum* Mill. (woolly burdock) represents a significant yet underutilized pharmacological resource. As a ruderal plant, it is a lesser-known representative of the genus; however, it remains a valuable source of bioactive compounds and secondary metabolites [7], offering a promising alternative for expanding the raw material base for anti-inflammatory phytotherapy.

In recent years, carbon dioxide (CO_2_) extraction has gained attention as an efficient method for obtaining phytopreparations enriched with biologically active constituents while avoiding the use of organic solvents and preserving thermolabile compounds [8]. Unlike traditional extraction methods, such as maceration or Soxhlet extraction, which often rely on high temperatures and polar solvents, the significance of applying this technology to *A. tomentosum* roots lies in the ability to selectively extract lipophilic and moderately polar bioactive compounds while minimizing degradation and solvent residues, which is critical for subsequent in vivo evaluation [9]. This green extraction approach supports the development of bioactive phytopreparations suitable for preclinical safety and pharmacological studies [10]. Accordingly, CO_2_-extract-based phytopreparations are increasingly regarded as promising candidates for biomedical research.

Our initial study demonstrated that a CO_2_ extract from *Arctium tomentosum* Mill. root exhibits antimicrobial activity against *Staphylococcus aureus*, *Staphylococcus epidermidis*, *Escherichia coli*, and *Candida albicans* [11]. Although the phytochemical profile of the previously studied subcritical CO_2_ extract was relatively modest [12], the extract still demonstrated pronounced antioxidant activity in FRAP and DPPH assays and showed protective effects in a murine model of metabolic disorder [13]. These results indicate that even a less chemically rich extract can exert significant in vivo biological activity, motivating the development of a CO_2_ extract with an ethanol co-solvent to enhance the recovery of bioactive constituents.

Cytotoxicity toward mononuclear lymphocytes was also evaluated using the MTT assay, together with acute and subacute toxicity studies conducted in rats [14]. However, those toxicity studies lacked weight measurements and histological examination of several vital organs, including the heart, lungs, spleen and gonads (testicles and ovaries). Given the therapeutic potential of this species and the need for a comprehensive safety profile to support its medical application, the present work was designed to comprehensively evaluate the acute and subacute toxicity of a CO_2_-extract-based phytopreparation from *Arctium tomentosum* Mill. roots (AT) in mice, as well as to investigate its systemic anti-inflammatory activity using xylene-induced ear edema and LPS-induced acute inflammation models.

## 2. Results

### 2.1. Chemical Composition of the CO_2_ Extract

The chemical composition of the CO_2_ extract obtained from *Arctium tomentosum* roots was analyzed using GC–MS. A total of 30 volatile and semi-volatile compounds were identified by comparing their mass spectra with the NIST 20 library and further verified by their calculated retention indices (RIs) relative to C_8_–C_40_ n-alkanes. Identification was considered reliable based on a match factor (SI) exceeding 850. Since a single quadrupole mass spectrometer was utilized, the identification was confirmed through spectral patterns and retention data rather than high-resolution accurate mass measurements.

The GC–MS analysis of the CO_2_ extract from *Arctium tomentosum* roots revealed a complex mixture of oxygen-containing organic compounds, cyclic acetals, aldehydes, and fatty acid derivatives (Table 1). The major constituents identified included 1,3-dioxolane-2-methanol (27.36%) and 1,1-diethoxyethane (acetaldehyde diethyl acetal, 12.63%). The presence of these acetals was critically interpreted as potential extraction artifacts resulting from the reaction of endogenous plant aldehydes with the ethanol co-solvent under supercritical conditions. Their potential biological role as membrane fluidity modulators is discussed in Section 3. Other significant components included 1,3-dioxolane derivatives (8.66%) and bicyclic monoterpenoid alcohols (7.73%).

**Figure 1:**
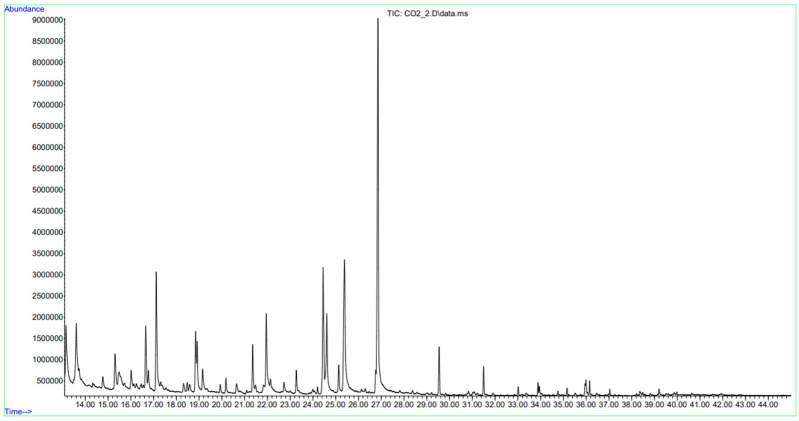
Total ion chromatogram (TIC) of the CO_2_ extract of *Arctium tomentosum* roots obtained by GC–MS analysis.

The total ion chromatogram (TIC) presented in Figure 1 illustrates the distribution and relative abundance of the identified constituents. The profile is characterized by several high-intensity peaks corresponding to the major oxygenated compounds, as well as a multitude of smaller peaks representing minor volatile metabolites. The well-resolved peaks and stable baseline of the TIC confirm the high efficiency of the CO_2_ extraction process in capturing a wide range of phytoconstituents from *Arctium tomentosum* roots.

GC–MS analysis of the CO_2_ extract from *Arctium tomentosum* roots revealed the presence of multiple volatile and semi-volatile constituents, mainly represented by oxygen-containing organic compounds. The dominant compounds identified in the extract included 1,3-dioxolane-2-methanol (27.36%), ethane, 1,1-diethoxy- (12.63%), 1,3-dioxolane derivatives (8.66%), and bicyclic monoterpenoid alcohol derivatives (7.73%). Minor components detected in smaller quantities included tetradecanal, hexadecanoic acid, and vinyl lauryl ether.

### 2.2. In Vivo Studies

#### 2.2.1. Study of the Acute Toxicity

All animals survived the 14-day experimental period, and no clinical signs of toxicity were observed. Body weight was monitored on Days 0, 7, and 14. Two-way repeated-measures ANOVA revealed a significant main effect of period (F(1.535, 18.42) = 469.3, *p* < 0.0001; ε = 0.7676), indicating a time-dependent increase in body weight. No significant effect of treatment (F(2, 12) = 0.2919, *p* = 0.7520) or interaction between period and treatment (F(3.07, 18.42) = 1.309, *p* = 0.3016) was detected.

Post hoc analysis using Dunnett’s test demonstrated a significant increase in body weight on Day 7 and Day 14 compared with Day 0 in all groups. In the baseline control group, body weight increased significantly on Day 7 (*p* = 0.0011) and Day 14 (*p* = 0.0002). Similarly, significant increases were observed in the AT 2000 mg/kg group (Day 7: *p* = 0.0030; Day 14: *p* = 0.0004) and in the AT 5000 mg/kg group (Day 7: *p* = 0.0026; Day 14: *p* = 0.0001). These findings confirm normal physiological growth over time and indicate that AT administration did not affect body weight dynamics during the study period (Table 2).

Relative organ weights were analyzed by one-way ANOVA after confirming normal distribution and homogeneity of variances (Brown–Forsythe and Bartlett’s tests, all *p* > 0.05). No significant differences among groups were observed for the heart (F(2, 12) = 1.021, *p* = 0.3896), liver (F(2, 12) = 0.8814, *p* = 0.4394), spleen (F(2, 12) = 2.000, *p* = 0.1780), lungs (F(2, 12) = 0.4259, *p* = 0.6627), or thymus (F(2, 12) = 2.160, *p* = 0.1580). A significant treatment effect was detected for kidney relative weight (F(2, 12) = 5.508, *p* = 0.0201). Dunnett’s post hoc test revealed a modest but statistically significant increase in the AT 2000 mg/kg group compared with the baseline control (mean difference = 0.08400; 95% CI: 0.00408 to 0.1639; *p* = 0.0397), whereas no significant difference was observed in the AT 5000 mg/kg group (mean difference = −0.01400; 95% CI: −0.09392 to 0.06592; *p* = 0.8716). Overall, AT administration did not produce consistent dose-dependent alterations in relative organ weights (Table 3).

Since no mortality, clinical signs of toxicity, or macroscopic alterations in the internal organs were detected, histological examination was not performed.

#### 2.2.2. Study of the Subacute Toxicity

During the 28-day treatment period, no mortality or clinical signs of toxicity were observed in any group. Body weight in both male and female mice increased progressively over time, consistent with normal physiological growth.

In males, two-way repeated measures ANOVA with Geisser–Greenhouse correction demonstrated a significant effect of period on body weight (F(1.301, 15.62) = 365.9, *p* < 0.0001). In contrast, neither treatment (F(2, 12) = 0.0896, *p* = 0.9149) nor the period × treatment interaction (F(2.603, 15.62) = 0.1281, *p* = 0.9235) was statistically significant. Post hoc Dunnett’s tests within each group showed significant increases in body weight at Days 7, 14, 21, and 28 compared with Day 0 (all adjusted *p* < 0.01), with comparable growth dynamics across the baseline and AT-treated groups. Similarly, in females, a significant effect of period was observed (F(2.091, 25.09) = 894.6, *p* < 0.0001). Treatment (F(2, 12) = 0.0292, *p* = 0.9713) and the period × treatment interaction (F(4.182, 25.09) = 0.4736, *p* = 0.7623) were not significant. Dunnett’s multiple comparisons test confirmed significant increases in body weight at all subsequent time points compared with Day 0 within each group (all adjusted *p* < 0.001), with no differences between the AT-treated and baseline animals at any time point (Table 4).

Two-way ANOVA revealed no significant effect of AT treatment (200 or 400 mg/kg) on the relative weights of the heart, liver, kidneys, spleen, lungs, or thymus (all *p* > 0.05). For the heart, treatment was not significant (F(2, 12) = 0.0257, *p* = 0.9747), and the sex × treatment interaction did not reach significance (*p* = 0.0719). A significant main effect of sex was observed (F(1, 12) = 21.39, *p* = 0.0006), with males exhibiting higher relative heart weight than females (mean difference = 0.0253; 95% CI: 0.0134–0.0373). For the liver, treatment had no effect (F(2, 12) = 0.0183, *p* = 0.9819), and no interaction was detected (*p* = 0.8741). A significant sex effect was present (F(1, 12) = 15.85, *p* = 0.0018), with females showing higher relative liver weights than males (mean difference = 0.3033; 95% CI: −0.4693 to −0.1373). For the kidneys, treatment was not significant (F(2, 12) = 0.0021, *p* = 0.9979), and no interaction was found (*p* = 0.4462). A strong sex effect was detected (F(1, 12) = 46.01, *p* < 0.0001), with males exhibiting higher relative kidney weights (mean difference = 0.1140; 95% CI: 0.0774–0.1506). For the spleen, treatment was not significant (F(2, 12) = 0.1826, *p* = 0.8354), whereas both sex (F(1, 12) = 43.02, *p* < 0.0001) and the sex × treatment interaction (F(2, 12) = 9.489, *p* = 0.0034) were significant. Post hoc analysis indicated that sex differences were more pronounced in the AT-treated groups, although no treatment-related effect was detected within each sex. For the lungs, treatment was not significant (F(2, 12) = 0.1034, *p* = 0.9026). A significant effect of sex (F(1, 12) = 20.57, *p* = 0.0007) and a significant sex × treatment interaction (F(2, 12) = 5.812, *p* = 0.0172) were observed, reflecting sex-dependent differences without a main treatment effect. For the thymus, treatment was not significant (F(2, 12) = 0.2581, *p* = 0.7767), while both sex (F(1,12) = 79.41, *p* < 0.0001) and the sex × treatment interaction (F(2, 12) = 9.882, *p* = 0.0029) were significant. Males exhibited higher relative thymus weights than females (mean difference = 0.0300; 95% CI: 0.0227–0.0373). Analysis of reproductive organs showed no treatment-related effects. In males, one-way ANOVA demonstrated no differences in the relative weight of gonads (testicles) among the groups (F(2, 12) = 0.2277, *p* = 0.7997). Variance homogeneity was confirmed (Brown–Forsythe *p* = 0.6492; Bartlett’s *p* = 0.7007). Dunnett’s test revealed no significant differences between the AT-treated groups and the baseline group. Similarly, in females, the relative weight of gonads (ovaries) did not differ between the groups (F(2, 12) = 2.177, *p* = 0.1560), with no heterogeneity of variances (Brown–Forsythe *p* = 0.3804; Bartlett’s *p* = 0.1772). Dunnett’s post hoc comparisons showed no significant differences versus baseline. Overall, these results demonstrate that AT administration at 200 and 400 mg/kg for 28 days does not affect relative organ weights. Observed differences were attributable to physiological sex-related variation rather than treatment-related toxicity (Table 5).

**Figure 2:**
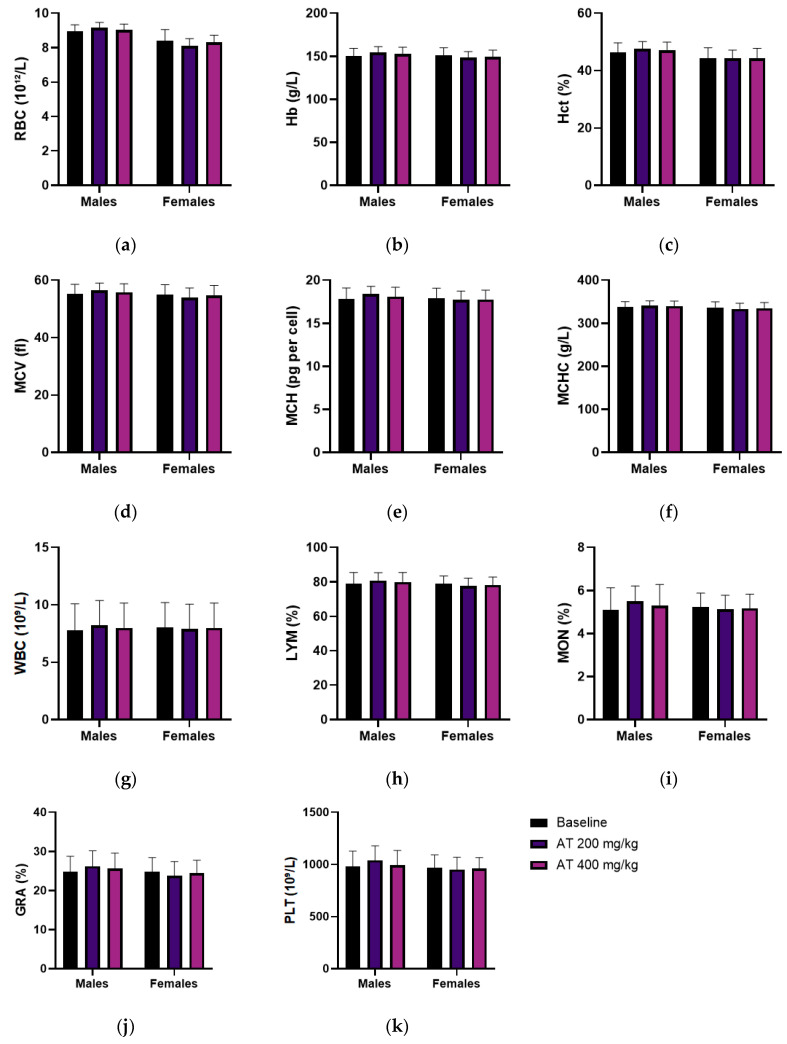
Hematological parameters of blood after the 28-day treatment period: (**a**) red blood cells; (**b**) hemoglobin; (**c**) hematocrit; (**d**) mean corpuscular volume; (**e**) mean corpuscular hemoglobin; (**f**) mean corpuscular hemoglobin concentration; (**g**) white blood cells; (**h**) lymphocytes; (**i**) monocytes; (**j**) granulocytes; (**k**) platelets. *n* = 10 per group (5 males and 5 females).

Two-way ANOVA showed no significant effect of AT treatment (200 or 400 mg/kg) on any hematological parameter (all *p* > 0.05), and no significant treatment × sex interactions were observed. A significant main effect of sex was detected for RBC (F(1, 24) = 24.89, *p* < 0.0001) and Hct (F(1, 24) = 5.749, *p* = 0.0246), with males exhibiting higher values than females. For RBC, sex accounted for 49.10% of the total variance. Post hoc analysis confirmed higher RBC levels in males at 200 and 400 mg/kg, whereas no differences were observed at baseline. No significant sex-related differences were found for Hb, MCV, MCH, MCHC, WBC, LYM, MON, GRA, or PLT (all *p* > 0.05). Overall, AT administration for 28 days did not induce hematological alterations. The observed differences were attributable to physiological sex-related variation rather than treatment effects (Figure 2a–k).

**Figure 3:**
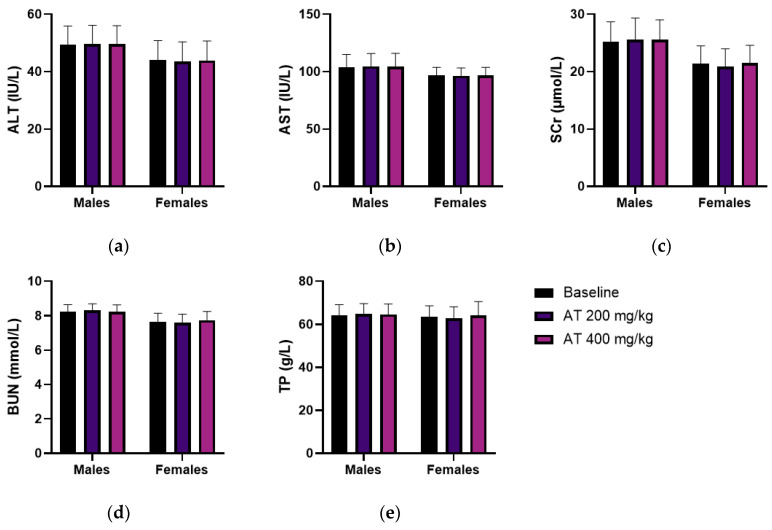
Biochemical parameters of plasma after the 28-day treatment period: (**a**) alanine aminotransferase; (**b**) aspartate transaminase; (**c**) serum creatinine; (**d**) blood urea nitrogen; (**e**) total protein. *n* = 10 per group (5 males and 5 females).

Two-way ANOVA revealed no significant effect of AT treatment (200 or 400 mg/kg) on ALT, AST, SCr, BUN, or TP (all *p* > 0.05), and no significant sex × treatment interactions were detected. A significant main effect of sex was observed for ALT (F(1, 24) = 5.506, *p* = 0.0275), AST (F(1, 24) = 5.038, *p* = 0.0343), SCr (F(1, 24) = 11.79, *p* = 0.0022), and BUN (F(1, 24) = 13.33, *p* = 0.0013), with males exhibiting higher mean values than females. Sex accounted for 18.65% of the total variation in ALT, 17.34% in AST, 32.81% in SCr, and 35.28% in BUN. Post hoc Bonferroni comparisons within each treatment group did not reveal consistent significant differences between sexes, except for BUN at 200 mg/kg (*p* = 0.0471), which was not accompanied by a significant interaction effect and therefore reflects normal biological variation rather than treatment-related modulation. Total protein (TP) was not influenced by sex, treatment, or their interaction (all *p* > 0.05). Overall, AT administration for 28 days did not induce hepatotoxic or nephrotoxic alterations, and the observed differences were attributable to physiological sex-related variation (Figure 3a–e).

**Figure 4:**
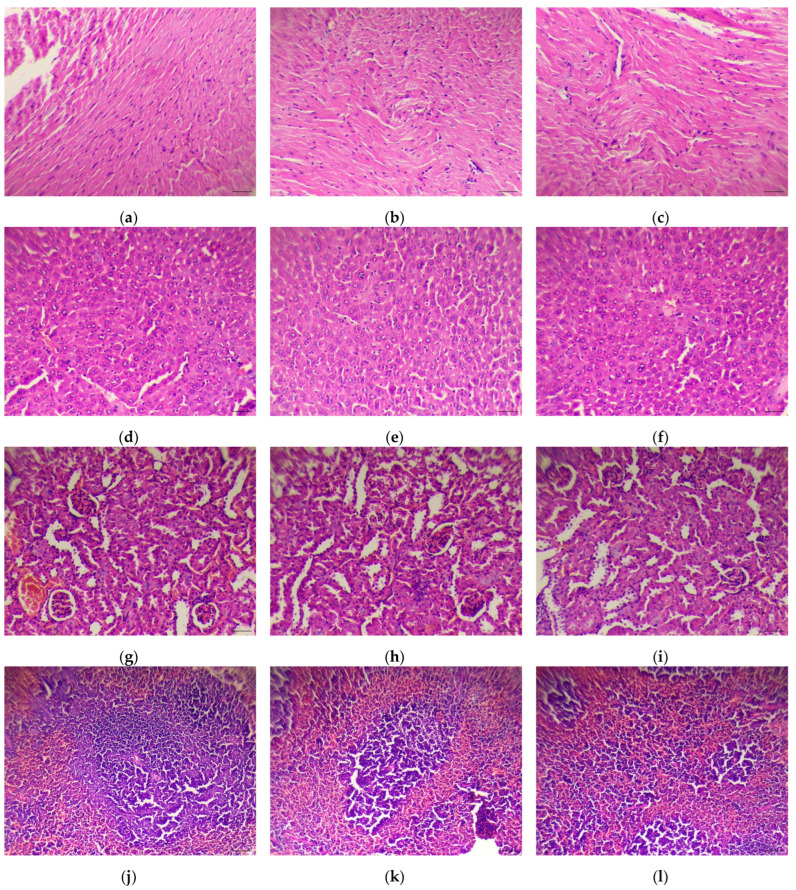

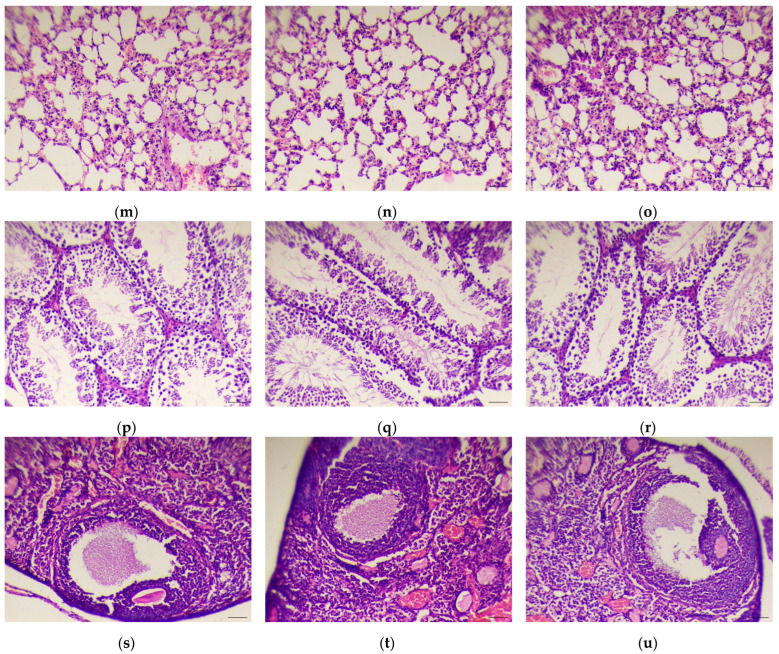
Histological structure of main internal organs after the 28-day treatment period: (**a**–**c**) heart; (**d**–**f**) liver; (**g**–**i**) kidneys; (**j**–**l**) spleen; (**m**–**o**) lungs; (**p**–**r**) testicles; (**s**–**u**) ovaries. Left panel—baseline; middle panel—AT 200 mg/kg; right panel—AT 400 mg/kg. H&E, 100 µm.

Macroscopic examination of internal organs, including the heart, liver, kidneys, spleen, lungs, thymus, and gonads (testes and ovaries), revealed no treatment-related gross pathological changes in any AT-treated group compared with the baseline control animals. All organs were anatomically intact and normally positioned within the body cavity. Their size, shape, color, and consistency were within physiological limits for the species and age. No signs of vascular congestion, hemorrhage, edema, focal discoloration, surface irregularities, nodular formations, or tissue fragility were observed. The liver exhibited a smooth capsule and homogeneous coloration; the kidneys showed preserved cortical–medullary distinction; the spleen and thymus were of normal appearance without enlargement or atrophy; the lungs were well aerated without visible consolidation; and reproductive organs were symmetrical and morphologically unremarkable. Overall, gross examination did not reveal any evidence of systemic toxicity associated with 28-day AT administration at the doses of 200 and 400 mg/kg (Figure 4a–u).

In summary, repeated oral administration of AT at doses up to 400 mg/kg for 28 days did not induce any clinical signs of toxicity, mortality, or significant changes in body weight, food consumption, or hematological and biochemical parameters. Macroscopic and histopathological examination of the major organs revealed no treatment-related alterations. These results indicate that AT is well tolerated and does not produce observable adverse effects under the conditions of this subacute toxicity study.

### 2.3. Study of the Anti-Inflammatory Effect of AT

#### 2.3.1. Anti-Inflammatory Effect in the Xylene-Induced Ear Edema Model

**Figure 5:**
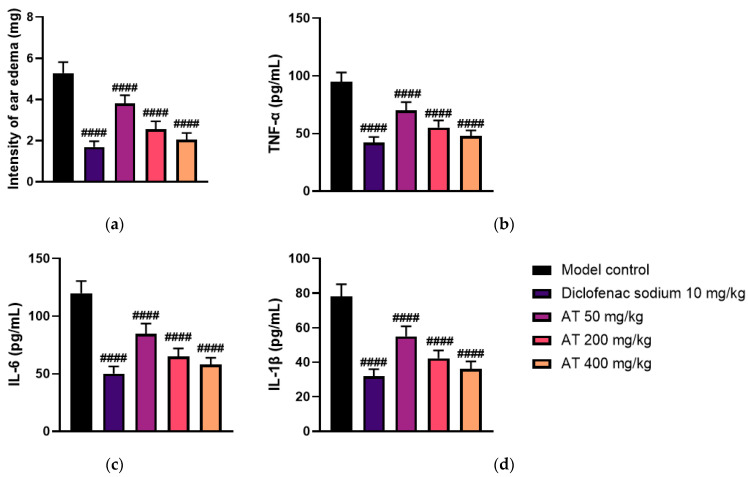
The study of the anti-inflammatory effect of AT on xylene-induced ear edema: (**a**) intensity of ear edema; (**b**) level of TNF-α; (**c**) level of IL-6; (**d**) level of IL-1β. *n* = 5 per group, #### *p* < 0.0001 vs. model control (one-way ANOVA followed by the Dunnett’s post-test).

One-way ANOVA demonstrated a significant effect of treatment on ear edema (F(4, 20) = 69.92, *p* < 0.0001, R^2^ = 0.9333), TNF-α (F(4, 20) = 56.66, *p* < 0.0001, R^2^ = 0.9189), IL-6 (F(4, 20) = 63.34, *p* < 0.0001, R^2^ = 0.9268), and IL-1β (F(4, 20) = 60.46, *p* < 0.0001, R^2^ = 0.9236). Homogeneity of variances was confirmed for all parameters by Brown–Forsythe and Bartlett’s tests (*p* > 0.05). Dunnett’s post hoc test showed that diclofenac produced the greatest reduction in ear edema (mean difference −3.60; 95% CI −4.26 to −2.94) and cytokine levels (TNF-α: −53.00; IL-6: −70.00; IL-1β: −46.20; all *p* < 0.0001) compared with the model control. AT demonstrated a dose-dependent effect, with higher doses (200 and 400 mg/kg) significantly reducing ear edema (−2.72 and −3.24, respectively) and cytokine concentrations (TNF-α: −40.00 and −47.00; IL-6: −55.00 and −62.00; IL-1β: −35.80 and −42.00; all *p* < 0.0001), approaching the efficacy of diclofenac, while 50 mg/kg showed moderate but significant effects (Figure 5a–d).

**Figure 6:**
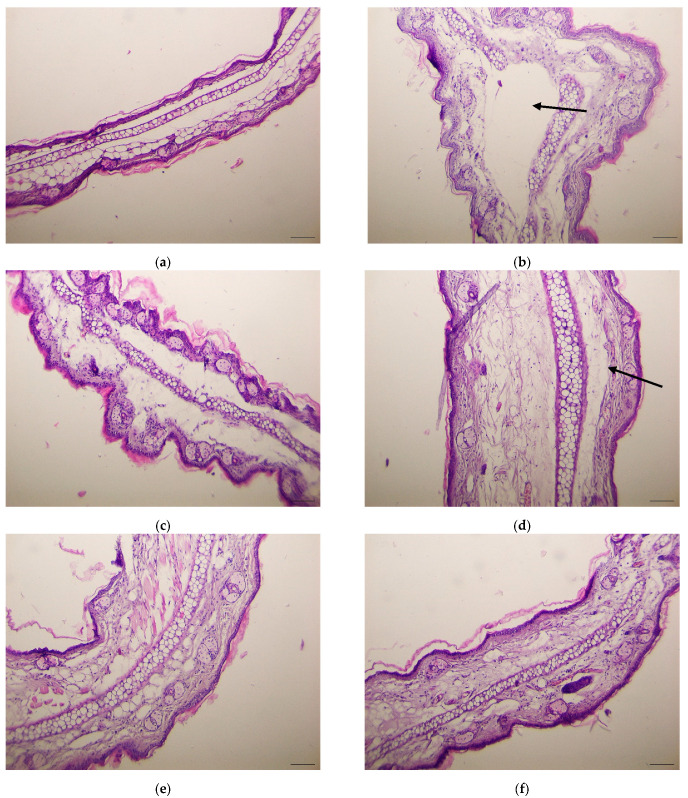
Histological structure of the ear tissue: (**a**) normal ear; (**b**) model control; (**c**) diclofenac sodium 10 mg/kg; (**d**) AT 50 mg/kg; (**e**) AT 200 mg/kg; (**f**) AT 400 mg/kg. Black arrow—interstitial edema. H&E, 200 µm.

Histological examination revealed clear differences among groups. Normal ear tissue (Figure 6a) showed an intact architecture with no signs of inflammation. In contrast, the model control group exhibited pronounced inflammatory changes, including marked dermal edema, vascular congestion, and dense infiltration of inflammatory cells (Figure 6b, black arrow). Treatment with diclofenac resulted in the most pronounced protective effect, substantially reducing edema and inflammatory cell infiltration, with the tissue structure approaching a normal morphology (Figure 6c). Among the AT-treated groups, 50 mg/kg produced moderate improvement with partial reduction in edema and infiltration (Figure 6d, black arrow), whereas 200 mg/kg (Figure 6e, black doted arrow) and 400 mg/kg (Figure 6f, black doted arrow) markedly attenuated histopathological abnormalities, showing reduced dermal thickness, fewer infiltrating leukocytes, and an overall tissue architecture closer to normal.

The histological findings corroborate the quantitative assessments of ear edema and pro-inflammatory cytokines, supporting a dose-dependent anti-inflammatory effect of AT comparable to diclofenac at higher doses (200 and 400 mg/kg).

#### 2.3.2. Anti-Inflammatory Effect in LPS-Induced Inflammation

**Figure 7:**
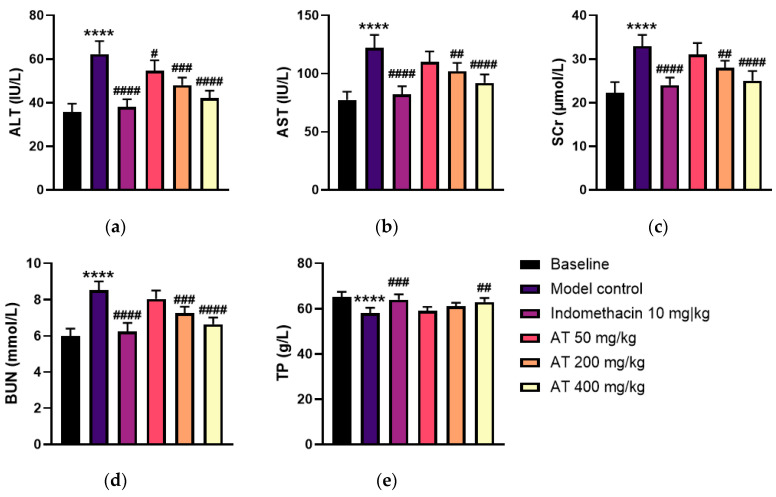
Biochemical parameters of plasma in LPS-induced inflammation: (**a**) alanine aminotransferase; (**b**) aspartate transaminase; (**c**) serum creatinine; (**d**) blood urea nitrogen; (**e**) total protein. *n* = 5 per group; **** *p* < 0.0001 vs. baseline; #### *p* < 0.0001, ### *p* < 0.001, ## *p* < 0.01, # *p* < 0.05 vs. model control (one-way ANOVA followed by the Dunnett’s post-test).

Analysis of serum biochemical parameters revealed significant differences among the treatment groups. For ALT, ANOVA indicated a significant effect of treatment (F(5, 24) = 28.58, *p* < 0.0001, Figure 7a), with Dunnett’s test showing significant reductions compared to the model control for baseline (−26.66, *p* < 0.0001), Indomethacin 10 mg/kg (−24.26, *p* < 0.0001), AT 50 mg/kg (−7.58, *p* = 0.0409), AT 200 mg/kg (−14.22, *p* = 0.0001), and AT 400 mg/kg (−20.22, *p* < 0.0001). AST levels were also significantly affected (F(5, 24) = 21.11, *p* < 0.0001, Figure 7b), with significant reductions for baseline (−44.72, *p* < 0.0001), Indomethacin 10 mg/kg (−40.02, *p* < 0.0001), AT 200 mg/kg (−20.00, *p* = 0.0038), and AT 400 mg/kg (−29.96, *p* < 0.0001), while AT 50 mg/kg did not differ significantly. Serum creatinine (SCr) showed significant treatment effects (F(5, 24) = 17.52, *p* < 0.0001, Figure 7c), with reductions for baseline (−10.74, *p* < 0.0001), Indomethacin 10 mg/kg (−8.94, *p* < 0.0001), AT 200 mg/kg (−5.00, *p* = 0.0074), and AT 400 mg/kg (−7.94, *p* < 0.0001), but no significant effect for AT 50 mg/kg. BUN was significantly decreased (F(5, 24) = 27.68, *p* < 0.0001, Figure 7d), with baseline (−2.54, *p* < 0.0001), Indomethacin 10 mg/kg (−2.28, *p* < 0.0001), AT 200 mg/kg (−1.28, *p* = 0.0004), and AT 400 mg/kg (−1.88, *p* < 0.0001) showing significant reductions, while AT 50 mg/kg had no significant effect. Total protein (TP) was significantly affected by treatment (F(5, 24) = 9.862, *p* < 0.0001, Figure 7e), with increases observed for baseline (7.28, *p* < 0.0001), Indomethacin 10 mg/kg (6.04, *p* = 0.0004), and AT 400 mg/kg (5.00, *p* = 0.0032), while AT 50 mg/kg and AT 200 mg/kg showed no significant change. Variance homogeneity was confirmed for all parameters by Brown–Forsythe and Bartlett’s tests.

**Figure 8:**
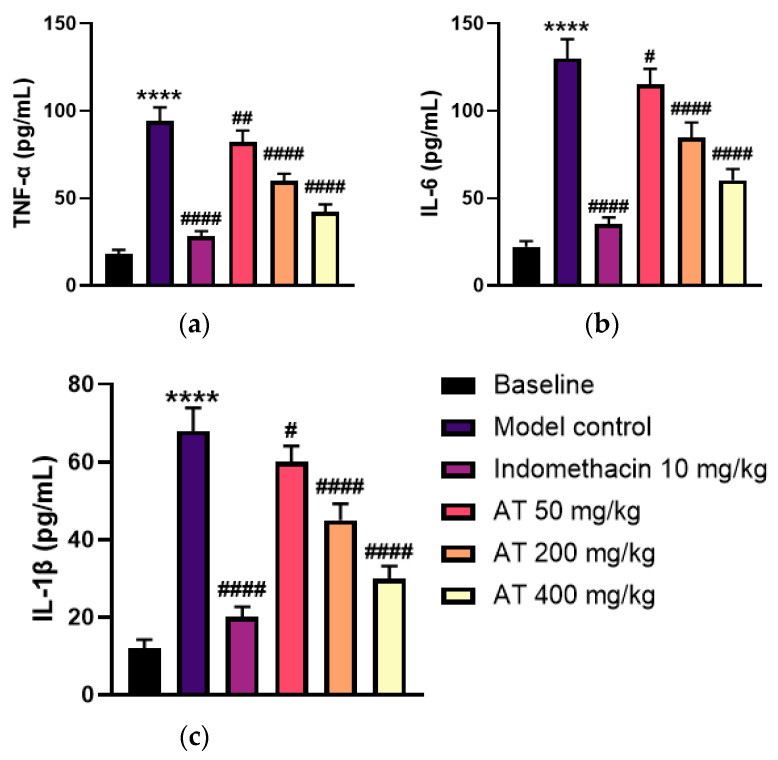
Serum cytokines in LPS-induced inflammation: (**a**) TNF-α; (**b**) IL-6; (**c**) IL-1β. *n* = 5 per group; **** *p* < 0.0001 vs. baseline; #### *p* < 0.0001, ## *p* < 0.001, # *p* < 0.05 vs. model control (one-way ANOVA followed by the Dunnett’s post-test).

Analysis of pro-inflammatory cytokines revealed significant differences among the treatment groups. TNF-α levels were significantly affected by treatment (F(5, 24) = 172.1, *p* < 0.0001, Figure 8a), with Dunnett’s test showing reductions compared to the model control for baseline (−76.04, *p* < 0.0001), Indomethacin 10 mg/kg (−66.02, *p* < 0.0001), AT 50 mg/kg (−12.00, *p* = 0.0049), AT 200 mg/kg (−34.04, *p* < 0.0001), and AT 400 mg/kg (−51.82, *p* < 0.0001). IL-6 levels were also significantly modulated (F(5, 24) = 160.2, *p* < 0.0001, Figure 8b), with significant reductions for baseline (−108.0, *p* < 0.0001), Indomethacin 10 mg/kg (−95.04, *p* < 0.0001), AT 50 mg/kg (−15.00, *p* = 0.0200), AT 200 mg/kg (−45.50, *p* < 0.0001), and AT 400 mg/kg (−69.74, *p* < 0.0001). Similarly, IL-1β showed significant treatment effects (F(5, 24) = 162.2, *p* < 0.0001, Figure 8c), with decreases for baseline (−55.92, *p* < 0.0001), Indomethacin 10 mg/kg (−47.96, *p* < 0.0001), AT 50 mg/kg (−7.96, *p* = 0.0155), AT 200 mg/kg (−22.94, *p* < 0.0001), and AT 400 mg/kg (−37.96, *p* < 0.0001). Variance homogeneity was confirmed for all cytokine parameters by Brown–Forsythe and Bartlett’s tests.

**Figure 9:**
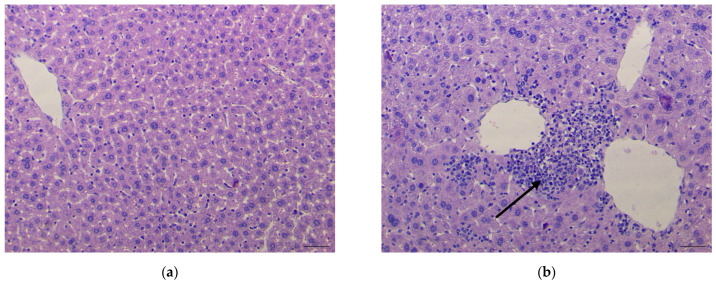

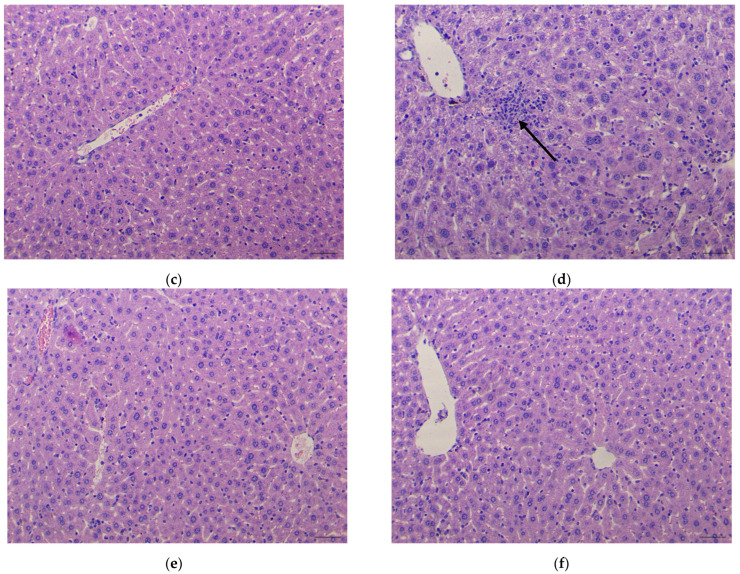
Histological structure of the liver: (**a**) baseline; (**b**) model control; (**c**) Indomethacin 10 mg/kg; (**d**) AT 50 mg/kg; (**e**) AT 200 mg/kg; (**f**) AT 400 mg/kg. Black arrow—inflammatory infiltrate. H&E, 100 µm.

The observed suppression of systemic pro-inflammatory cytokines was further corroborated by histopathological analysis of the liver (Figure 9).

The liver of the baseline group (Figure 9a) maintains a pristine histological structure, whereas the model control (Figure 9b) exhibits severe disruption of the hepatic cords accompanied by dense clusters of inflammatory cells (indicated by the black arrow). Treatment with the reference drug, Indomethacin (Figure 9c), effectively preserves tissue integrity, a trend mirrored by the AT-treated groups in a dose-dependent manner. At the 50 mg/kg dose (Figure 9d), although there is a significant reduction in TNF-α, residual focal accumulations of inflammatory cells remain visible, reflecting the lower degree of cytokine suppression compared to higher dosages. In contrast, the AT 200 mg/kg (Figure 9e) and 400 mg/kg (Figure 9f) groups demonstrate a robust hepatoprotective effect, with the 400 mg/kg dose showing nearly complete restoration of the sinusoidal spaces and a total absence of degenerative changes, thereby providing visual confirmation that the CO_2_ extract limits structural organ damage by effectively quenching the LPS-induced cytokine-mediated inflammatory cascade.

## 3. Discussion

### 3.1. Chemical Composition of the CO_2_ Extract and Its Biological Relevance

GC–MS analysis demonstrated that the CO_2_ extract of *Arctium tomentosum* roots contains a complex mixture of oxygen-containing organic compounds, including cyclic acetals, aldehydes, and fatty acid derivatives. Among them, 1,3-dioxolane-2-methanol and ethane, 1,1-diethoxy- represented the major constituents of the extract.

The chemical profile of our CO_2_ extract exhibits distinct peculiarities compared to traditional extracts of *A. tomentosum*. While previous studies using HPLC and polar solvents have focused on the lignan content, specifically arctiin and arctigenin [15], or the phenolic capacity of roots influenced by harvest time [16], our CO_2_ extraction process preferentially recovers a lipophilic fraction dominated by oxygenated organic compounds and cyclic acetals (~49%). This specific “CO_2_ fingerprint” preserves volatile and thermolabile components that are typically underrepresented in the hydro-alcoholic or digested extracts reported in the existing literature [17].

It should be noted that while GC–MS analysis successfully characterized this lipophilic fraction, the method is inherently limited in its ability to detect non-volatile markers typical for the *Arctium* genus, such as the lignans arctiin and arctigenin, or phenolic acids like chlorogenic acid [18]. These compounds possess potent anti-inflammatory properties and are usually identified via HPLC/UHPLC in polar extracts [19,20]. Our findings complement existing knowledge by providing a detailed profile of the volatile constituents, although future studies focusing on the quantitative standardization of both fractions will be essential for the further development of this phytopreparation.

Furthermore, it is important to critically interpret the presence of 1,3-dioxolane-2-methanol and 1,1-diethoxyethane. These compounds are likely extraction artifacts resulting from the acetalization of endogenous plant aldehydes with the ethanol co-solvent under supercritical conditions [21,22]. Although they may not naturally occur in the plant tissue, their presence is of high pharmacological importance. Rather than acting as primary anti-inflammatory agents, these lipophilic acetals may function as membrane fluidity modulators [23]. By altering the microviscosity of the cell membrane, they potentially enhance the bioavailability and intracellular penetration of other bioactive constituents, such as lignans or phenolic compounds [24]. This synergistic “carrier” effect could explain the pronounced systemic anti-inflammatory response observed in our in vivo models at low dosages [25].

### 3.2. Acute and Subacute Toxicity of AT

The present acute toxicity study demonstrated that AT did not induce mortality, clinical signs of toxicity, or alterations in body weight and relative organ weights at the doses of 2000 and 5000 mg/kg, indicating a favorable safety profile in accordance with OECD guideline criteria (no observed adverse effects) for acute oral toxicity. The isolated increase in kidney relative weight at 2000 mg/kg, without a corresponding change at 5000 mg/kg, likely reflects normal biological variability rather than a toxic effect. No clinical signs, mortality, or macroscopic organ alterations were observed, supporting the absence of adverse effects. Comparable findings have been reported in other rodent acute toxicity assessments, where high doses (e.g., ≥2000–5000 mg/kg) produced no significant impact on body weight, organ coefficients, or clinical parameters, supporting the classification of such substances as practically nontoxic in vivo (e.g., anthraquinone having an LD_50_ > 5000 mg/kg and no adverse systemic effects) [26]. Similarly, acute oral toxicity studies of Karanjin indicated uniform weight gain and lack of systemic toxicity at doses up to 2000 mg/kg, reflecting preserved energy balance and physiological homeostasis in test animals [27]. Another acute toxicity assessment of almond hull powders showed no detrimental changes in mortality, body weight, food intake, or organ-to-body-weight ratios even at doses up to 5000 mg/kg, further corroborating the absence of toxicological effects under the tested conditions [28].

The results of the 28-day subacute toxicity study indicate that administration of AT at doses of 200 and 400 mg/kg did not induce mortality, observable clinical signs of toxicity, or adverse effects on body weight in male and female mice. The gradual increase in body weight observed in all groups is consistent with normal growth patterns and aligns with previous reports that nontoxic plant-derived compounds do not alter growth parameters over subacute exposure periods [29,30]. Evaluation of relative organ weights revealed no statistically significant effects of AT treatment on the heart, liver, kidneys, spleen, lungs, thymus, and gonads in either sex. Organ weight is a sensitive indicator of potential organ-specific toxicity, and our findings suggest that AT does not produce hypertrophy, atrophy, or other organ-specific adverse effects [31]. The significant differences in organ weights between sexes are consistent with established physiological differences in mice, including larger livers and gonads in females and heavier hearts, kidneys, spleens, lungs, and thymuses in males [32]. These sex-related differences were maintained in all experimental groups, further confirming the absence of treatment-related toxicity. Further observed sex differences, with males exhibiting higher RBC, Hb, Hct, MCV, MCH, MCHC, and PLT values compared to females, are consistent with known physiological variations in rodents and reflect normal sexual dimorphism in hematology [33]. Among leukocyte populations, significant sex differences in LYM and GRA were also observed, whereas total WBC and MON counts were comparable between sexes. These findings are in line with prior reports on baseline hematological profiles in male and female mice [34]. The serum biochemical analysis demonstrated that subacute administration of AT at doses of 200 and 400 mg/kg for 28 days did not produce significant alterations in liver or kidney function markers, including ALT, AST, SCr, BUN and TP. Minor sex × treatment interactions were observed for BUN; however, the effect was modest and of no clinical relevance [35]. Gross examination remains an essential component of subacute toxicity assessments, as visible changes in organ size, color, or texture may reflect underlying pathological processes [36]. The absence of macroscopic and histopathological alterations in AT-treated animals indicates that repeated oral administration of AT at doses of 200 and 400 mg/kg for 28 days does not induce structural toxicity in internal organs.

Histopathological analysis is considered a gold standard for detecting subtle or early toxic effects that may not be reflected in clinical observations or biochemical parameters [37]. The preservation of normal tissue architecture in the liver, kidneys, heart, lungs, spleen, thymus, and gonads indicates that AT does not induce inflammatory, degenerative, or necrotic changes under the tested conditions. Importantly, the lack of hepatic and renal histological alterations is consistent with the stable serum ALT, AST, SCr, and BUN levels observed in this study, further supporting the absence of hepatotoxic or nephrotoxic effects. These findings are in agreement with previous reports evaluating the safety of plant-derived or bioactive compounds in 28-day repeated-dose studies, where no treatment-related histopathological changes were observed at comparable dose ranges [38,39]. Collectively, the morphological data corroborate the hematological and biochemical findings and confirm that AT is well tolerated in both male and female mice, without evidence of structural organ damage following subacute administration.

Based on the absence of mortality at the highest tested dose, the LD_50_ of AT was determined to be >5000 mg/kg, allowing its classification as practically nontoxic (GHS Category 5) [40].

### 3.3. Anti-Inflammatory Effect of AT

#### 3.3.1. Xylene-Induced Ear Edema

The results obtained during the study of the anti-inflammatory effect of AT on the xylene-induced ear edema model demonstrate a robust anti-inflammatory effect of AT, evidenced by significant suppression of both local inflammatory response and systemic pro-inflammatory mediators. This pharmacological efficacy is likely rooted in the unique chemical composition of the CO_2_ extract (Table 1), which is characterized by a high concentration of oxygenated lipophilic compounds. The significant suppression of pro-inflammatory cytokines (TNF-α, IL-6, IL-1β) and local edema observed in this study aligns with established inflammatory pathways in acute inflammatory responses. Pro-inflammatory cytokines such as TNF-α, IL-6, and IL-1β are central drivers of the inflammatory cascade. In the xylene-induced model, which is characterized by rapid vasodilation and plasma extravasation, the identified components of AT, particularly 1,3-dioxolane-2-methanol and ethane, 1,1-diethoxy-, may play a crucial role due to their high penetrability and potential to modulate early mediator release. Elevated TNF-α and IL-1β promote endothelial activation and leukocyte infiltration, contributing to tissue swelling and pain, whereas IL-6 sustains the acute phase response and bridges innate and adaptive immunity. The presence of bicyclic terpenoids, specifically Bicyclo[3.1.1]heptan-3-ol derivative (7.73%), and long-chain fatty acids, like hexadecanoic acid (0.45%), further enhances the biological plausibility of the observed effects. These compounds are known to stabilize lysosomal membranes and interfere with the arachidonic acid cascade, thereby reducing the production of prostaglandins and leukotrienes that drive the exudative phase of xylene-induced edema. Consistent with these mechanisms, interventions that reduce TNF-α, IL-6, and IL-1β levels have demonstrated anti-inflammatory efficacy in diverse experimental and clinical settings [41,42]. For example, systematic reviews highlight the pathogenic roles of IL-6 and IL-1β in cardiovascular diseases, where elevated cytokine concentrations are associated with adverse outcomes, suggesting that targeted modulation of these cytokines can yield therapeutic benefit [43]. In addition, pro-inflammatory cytokines such as TNF-α and IL-6 are implicated in sustaining skin inflammation and neuro-inflammatory processes, further underscoring their importance as therapeutic targets [44]. The dose-dependent pattern of TNF-α, IL-6, and IL-1β inhibition by AT in the current model suggests that AT may modulate upstream signaling pathways that converge on these cytokines, potentially involving common transcriptional regulators like NF-κB (a key node in pro-inflammatory signaling) and CREB-dependent mechanisms that coordinate cytokine expression [45,46].

Furthermore, the specific “green” extraction profile, which avoids thermal degradation of these sensitive oxygenated molecules, ensures that the native anti-inflammatory synergy of the *Arctium tomentosum* roots is preserved. The histological findings corroborate the dose-dependent anti-inflammatory efficacy of AT observed in edema and cytokine assays. These results are consistent with prior studies demonstrating that suppression of TNF-α, IL-6, and IL-1β correlates with improved histopathological outcomes in models of acute inflammation [47,48,49]. The concordance between reduced cytokine levels and attenuated tissue damage underscores the therapeutic potential of AT as a potent anti-inflammatory agent that may be capable of both biochemical and morphological modulation of inflammation.

#### 3.3.2. LPS-Induced Inflammation

The results obtained during the study of the anti-inflammatory effect of AT in LPS-induced inflammation demonstrate a robust systemic response. The mechanism behind this protective effect likely involves the suppression of the TLR4-mediated signaling pathway, which is triggered by LPS. The observed reductions in ALT and AST indicate hepatoprotective effects, as AT likely mitigates the inflammatory infiltration and oxidative stress within the hepatic parenchyma. This is consistent with prior studies demonstrating the protective effects of anti-inflammatory and antioxidant agents against liver enzyme elevation [50,51].

Significant decreases in SCr and BUN suggest a nephroprotective effect of the treatments, aligning with reports that certain bioactive compounds can mitigate kidney injury markers under experimental stress [52]. The increase in TP, particularly with higher doses, may reflect improved liver synthetic function or reduced protein catabolism, as reported in rodent models exposed to hepatotoxic or nephrotoxic challenges [53,54].

The significant reductions in TNF-α, IL-6, and IL-1β indicate a potent anti-inflammatory effect. Molecularly, the AT extract appears to act as an inhibitor of the NF-κB signaling cascade, a master regulator of the inflammatory response. By preventing the overproduction of these primary cytokines, AT interrupts the feed-forward loop of systemic inflammation that typically leads to multi-organ failure in LPS models. These results align with previous studies showing that both natural bioactive compounds and standard anti-inflammatory drugs can downregulate key pro-inflammatory cytokines in rodent models [55,56].

The dose-dependent hepatoprotective and nephroprotective effects are directly linked to this cytokine suppression, as TNF-α and IL-1β are known to induce apoptosis in hepatocytes and renal tubular cells [57,58,59,60]. Thus, AT demonstrated multi-organ protective effects, significantly improving liver and kidney function markers (ALT, AST, sCr, BUN, TP) and reducing systemic inflammation by suppressing pro-inflammatory cytokines (TNF-α, IL-6, IL-1β) in a dose-dependent manner. These results highlight its potential as a therapeutic agent with hepatoprotective, nephroprotective, and anti-inflammatory properties, driven by its ability to modulate systemic inflammatory signaling.

## 4. Materials and Methods

### 4.1. Plant Material and Extraction Process

Roots of *Arctium tomentosum* Mill. were collected from the Aksai Gorge of the Northern Tian Shan Mountains (Almaty city, Kazakhstan). A voucher specimen (No. 0002321) was deposited at the Institute of Botany and Phytointroduction. The roots were air-dried at ambient temperature, milled to a fine powder, and stored at room temperature in the dark until extraction.

The extraction of *Arctium tomentosum* roots was performed using an SFE-5000 apparatus (Thar Technologies, Pittsburgh, PA, USA). To recover both lipophilic and moderately polar bioactive constituents in a single step, CO_2_ with 5% (*v*/*v*) ethanol was employed. In each cycle, 500 g of powdered material was loaded, and the process was carried out at 50 °C and 40 MPa with a flow rate of 15 g/min for 120 min. These parameters were selected based on established optimization protocols for the recovery of bioactive compounds from plant matrices [61,62,63]. After removing the solvent under reduced pressure, a thick, ointment-like extract with a characteristic odor was obtained. The final yield was 1.8% (*w*/*w*), providing approximately 9 g of extract per 500 g of dry root powder.

### 4.2. GC–MS Analysis of the CO_2_ Extract

The chemical composition of the CO_2_ extract from *Arctium tomentosum* roots was analyzed using gas chromatography–mass spectrometry (GC–MS) on an Agilent 7890A gas chromatograph coupled with a 5975C mass selective detector (Agilent Technologies, Santa Clara, CA, USA). Separation was carried out on a DB-5MS capillary column (30 m × 0.25 mm × 0.25 μm). Helium was used as the carrier gas at a constant flow rate of 1 mL/min. The injection volume was 0.5 μL with splitless mode. The injector temperature was 280 °C. The oven temperature program was set from 40 °C to 200 °C at 5 °C/min and then to 280 °C at 10 °C/min. Mass spectra were recorded in SCAN mode (*m*/*z* 34–850).

Identification of the constituents was performed by comparing the obtained mass spectra with those stored in the NIST 20 and Wiley spectral libraries. To ensure the reliability of the identification, experimental linear retention indices (RIs) were calculated for each peak using a homologous series of n-alkanes (C_8_–C_40_) analyzed under the same chromatographic conditions. The identification was confirmed by comparing the experimental RI values with the literature data (NIST Chemistry WebBook, NIST Standard Reference Database Number 69, 2025/2026 Update). Only compounds with a library match factor (SI) greater than 850 were considered identified. The relative content of the components was expressed as a percentage of the total peak area, calculated using the area normalization method without the use of internal standards or response correction factors.

### 4.3. Animal Housing, Welfare, and Ethical Approval

All animals were maintained under standard conditions (22 ± 2 °C, 50 ± 10% humidity, 12 h light/dark), housed five per cage with ad libitum access to chow and water, and acclimatized for 7 days. Procedures complied with Directive 2010/63/EU and were approved by the Ethical Committee of JSC “Scientific Center for Anti-Infectious Drugs” (No. 25/9, 15 September 2025), in accordance with the Guide for the Care and Use of Laboratory Animals and ARRIVE guidelines. The animals were obtained from a licensed facility; they were healthy, pathogen-free, unmodified, and not previously used. The study included randomization, standardized housing/handling, and blinding where applicable. Confounders were minimized by consistent conditions, randomized cage placement, and uniform timing. No inclusion/exclusion criteria were applied, and no data were excluded. Welfare was monitored with predefined humane endpoints; no adverse events occurred.

### 4.4. Toxicity Studies

#### 4.4.1. Acute Toxicity Test

The acute oral toxicity study was conducted in accordance with the Organization for Economic Cooperation and Development (OECD) Guidelines for the Testing of Chemicals, Test No. 420: Acute Oral Toxicity—Fixed Dose Procedure [64]. Healthy male Swiss albino mice (25–35 g, 8–10 weeks old) were randomly allocated into three experimental groups (five animals per group): 1—baseline control, 2—AT 2000 mg/kg, and 3—AT 5000 mg/kg. Following a single oral administration, the animals were closely observed for clinical signs of toxicity and mortality during the first 4 h, periodically during the first 24 h, and daily thereafter for a total observation period of 14 days. Body weight was recorded once a week. Primary outcome measures included clinical signs of toxicity (e.g., changes in behavior, posture, locomotion, and general appearance), mortality, and body weight changes over the 14-day observation period. At the end of the observation period, all animals were euthanized, and a gross necropsy was performed to assess possible treatment-related pathological changes.

#### 4.4.2. Dose Selection and Human Equivalent Dose (HED) Calculation

The doses for the subacute toxicity study (200 and 400 mg/kg) were selected based on the results of the acute toxicity screening and preliminary dose-range finding studies. To ensure the clinical relevance of these doses, the Human Equivalent Dose (HED) was calculated using the body surface area (BSA) normalization method:

HED (mg/kg) = Animal Dose (mg/kg) × Animal K_m_/Human K_m_, (1)

where Animal K_m_ and Human K_m_ are the factor constants (3 for mice and 37 for humans) used to normalize doses across species.

Using this formula, the 400 mg/kg dose in mice translates to an HED of approximately 32.4 mg/kg, which corresponds to an intake of ~1.94 g/day for a 60 kg human. This calculation provides a pharmacological justification for the doses used in the 28-day repeated-dose study.

#### 4.4.3. Subacute Toxicity Test

The subacute toxicity study was performed in compliance with OECD Guidelines for the Testing of Chemicals, Test No. 407: Repeated Dose 28-Day Oral Toxicity Study in Rodents [65]. Healthy male and female Swiss albino mice (25–35 g, 8–10 weeks old) were randomly divided into three groups (five males and five females per group): 1—baseline control, 2—AT 200 mg/kg, and 3—AT 400 mg/kg. The test substance was administered orally once daily for 28 consecutive days. The animals were monitored daily for general clinical condition, behavioral changes, and signs of systemic toxicity. Body weight and food consumption were recorded weekly. At the end of the treatment period, blood samples were collected for hematological and biochemical analyses. The animals were then euthanized, and a complete gross necropsy was conducted. Internal organs (hearts, livers, kidneys, spleen, lungs, thymuses and gonads) were excised, weighed to determine relative organ weights, and examined for macroscopic alterations. For histological examination, the excised organs (hearts, livers, kidneys, spleens, lungs and gonads) were immediately fixed in 10% neutral buffered formalin for 24–48 h. The fixed tissues were then dehydrated through a graded series of ethanol, cleared in xylene, and embedded in paraffin wax. Sections of 4–5 μm thickness were cut using a microtome and mounted on glass slides. The slides were stained with hematoxylin and eosin (H&E) following standard protocols. Histopathological evaluation was performed under a light microscope at magnifications of 10× for spleens and 20× for hearts, livers, kidneys, lungs, testicles and ovaries to assess structural integrity and the presence of cellular degeneration, necrosis, inflammation, or other morphological changes. Findings were documented and compared across the baseline control and AT-treated groups.

### 4.5. Murine Models of Inflammation

#### 4.5.1. Xylene-Induced Ear Edema Model

Although the xylene-induced ear edema model is often used for topical formulations, it is also a validated method for evaluating systemic anti-inflammatory activity following oral administration, as the reduction in edema reflects inhibition of inflammatory mediators and vascular permeability. The study was conducted according to the protocols described by Singsai et al. [66] and Freitas et al., with slight modifications [67]. Healthy male Swiss albino mice (25–30 g, 6–8 weeks old) were randomly divided into five groups (five animals per group): 1—xylene only (model control), 2—xylene + diclofenac sodium 10 mg/kg, 3—xylene + AT 50 mg/kg, 4—xylene + AT 200 mg/kg, and 5—xylene + AT 400 mg/kg. They received the assigned treatment once daily for 7 consecutive days. One hour after the last administration, 0.03 mL of xylene was applied to the anterior and posterior surfaces of the left ear of each mouse. After thirty minutes, the mice were euthanized by deep isoflurane narcosis, and both ears were removed and weighed. Blood samples were collected for subsequent evaluation of biochemical parameters and pro-inflammatory cytokines. The right ear was considered the control. The intensity of edema was calculated according to the following formula:

Intensity of edema = E_left_ − E_right_, (2)

where E_left_ and E_right_ represent the weight of the left ear and the right ear, respectively.

For histological analysis, the excised ears were immediately fixed in 10% neutral buffered formalin for 24 h. The fixed tissues were dehydrated through graded ethanol, cleared in xylene, and embedded in paraffin. Sections of 4–5 μm thickness were cut using a microtome (Leica Biosystems, Wetzlar, Germany) and mounted on glass slides. The slides were stained with hematoxylin and eosin (H&E) to evaluate histopathological changes, including epidermal thickness, inflammatory cell infiltration, edema, and vascular congestion. Observations were performed under a light microscope (Carl Zeiss AG, Oberkochen, Germany) at magnification of 10×. Histological findings were compared between the treated and control groups to assess the anti-inflammatory effect of AT.

#### 4.5.2. LPS-Induced Inflammation Model

The lipopolysaccharide (LPS)-induced inflammation model was used to evaluate the anti-inflammatory activity of AT under conditions of acute systemic inflammatory response. Healthy male Swiss albino mice (25–30 g, 6–8 weeks old) were randomly divided into six groups (five animals per group): 1—baseline control, 2—LPS only (model control), 3—LPS + Indomethacin (10 mg/kg), 4—LPS + AT 50 mg/kg, 5—LPS + AT 200 mg/kg, and 6—LPS + AT 400 mg/kg. The animals received the assigned treatment once daily for 7 consecutive days prior to LPS challenge. Acute inflammation was induced by intraperitoneal injection of LPS (5 mg/kg), administered 1 h after the last assigned treatment [68]. The animals were monitored for general clinical condition following LPS administration. Four hours after LPS injection, the mice were euthanized under deep isoflurane anesthesia. Blood samples were collected for subsequent evaluation of biochemical parameters and pro-inflammatory cytokines.

### 4.6. Statistical Analysis

All data are presented as mean ± standard deviation. Statistical analyses were performed using GraphPad Prism 10.6.1 (GraphPad Software, San Diego, CA, USA). One-way or two-way analysis of variance (ANOVA) was performed, followed by Dunnett’s test or the Bonferroni post hoc test for multiple comparisons, where appropriate. Normality and homogeneity of variance were assessed using Shapiro–Wilk and Levene’s tests. Differences were considered statistically significant at *p* < 0.05.

## 5. Conclusions

The CO_2_ extraction of *Arctium tomentosum* roots, enhanced with an ethanol co-solvent, yielded a unique phytocomplex characterized by a specific chemical fingerprint. GC–MS analysis identified a high concentration of oxygen-containing organic compounds, dominated by cyclic acetals (specifically 1,3-dioxolane-2-methanol and 1,1-diethoxyethane) and fatty aldehydes. This distinctive composition, preserved by the mild extraction conditions, is considered a key factor underlying the extract’s biological potency, potentially through the modulation of membrane fluidity and enhanced bioavailability of active metabolites.

While the present study provides a detailed characterization of the lipophilic fraction of the *Arctium tomentosum* root CO_2_ extract, we acknowledge that further research focusing on the quantitative standardization of major lignans (arctiin and arctigenin) via HPLC will be necessary to ensure the reproducibility and quality control of this phytopreparation for clinical applications.

Our findings demonstrate that the CO_2_ extract (AT) exerts potent systemic anti-inflammatory and organ-protective effects by significantly suppressing the production of pro-inflammatory cytokines (TNF-α, IL-6, and IL-1β). This modulation effectively mitigates LPS-induced liver and kidney injury, likely through the stabilization of cellular membranes and the limitation of the cytokine-mediated inflammatory cascade. Furthermore, AT demonstrated a favorable safety profile (LD_50_ > 5000 mg/kg), classifying it as practically nontoxic.

Collectively, these results support the further investigation of the *A. tomentosum* CO_2_ extract as a promising therapeutic candidate for conditions involving systemic inflammation and oxidative stress.

## Figures and Tables

**Table 1:** Chemical composition of the CO_2_ extract from *Arctium tomentosum* roots identified by GC–MS analysis.

RT (min)	Compound Name (IUPAC/Common)	RI (exp)	RI (lit)	SI (Match)	Main Fragments (*m*/*z*)	Relative Area (%)
13.61	1,1-Diethoxyethane	718	715	912	47, 73, 103	5.49
15.31	(S)-2,3-Dihydroxypropanal	824	820	885	43, 61, 90	2.52
16.02	Acetic acid, methoxy-, ethyl ester	865	862	890	45, 59, 89	1.25
16.66	1,3-Dioxolane-2-methanol	895	890	904	43, 61, 103	4.09
17.12	Bicyclo[3.1.1]heptan-3-ol, 6,6-dimethyl-2-methylene	1068	1065	925	91, 107, 121	7.73
18.85	Benzoic acid, 1-methylethyl ester	1145	1142	910	105, 123, 164	3.50
21.36	2-Methoxy-1,3-dioxolane	935	932	875	59, 73, 103	2.72
22.73	Propane, 1-(1-ethoxyethoxy)-	955	950	880	45, 73, 89	0.71
23.27	Ethanol, 2,2′-[1,2-ethanediylbis(oxy)]bis-, diacetate	1655	1652	895	43, 87, 117	1.40
24.45	1,3-Dioxolane, 2-(1-methylethyl)-	978	975	902	43, 73, 115	8.66
25.39	1,1-Diethoxyethane	718	715	920	47, 73, 103	12.63
26.76	Vinyl lauryl ether	1575	1570	890	43, 57, 184	0.92
26.86	1,3-Dioxolane-2-methanol	895	890	930	43, 61, 103	27.36
29.55	Tetradecanal	1615	1612	915	43, 57, 82, 212	2.30
31.50	Hexadecane, 1-(ethenyloxy)-	1775	1772	885	43, 57, 240	1.30
35.96	Hexadecanoic acid	1972	1968	945	43, 73, 256	0.45
39.21	4-Octadecenal	2015	2010	870	41, 55, 83, 266	0.20

**Table 2:** Body weight during the 14-day observation period, g.

Group	Day 0	Day 7	Day 14
Baseline	29.2 ± 2.0	31.7 ± 1.9	33.7 ± 2.0
AT 2000 mg/kg	28.5 ± 2.2	30.3 ± 2.5	32.5 ± 2.3
AT 5000 mg/kg	29.0 ± 2.2	31.0 ± 2.6	33.5 ± 2.6

Data are presented as mean ± SD, *n* = 5 per group.

**Table 3:** Relative weight of organs after euthanasia, %.

Group	Heart	Liver	Kidneys	Spleen	Lungs	Thymus
Baseline	0.51 ± 0.01	5.08 ± 0.10	1.21 ± 0.07	0.42 ± 0.01	0.72 ± 0.02	0.16 ± 0.01
AT 2000 mg/kg	0.53 ± 0.02	5.18 ± 0.21	1.30 ± 0.03	0.44 ± 0.02	0.73 ± 0.03	0.17 ± 0.01
AT 5000 mg/kg	0.53 ± 0.03	5.24 ± 0.24	1.20 ± 0.04	0.44 ± 0.02	0.74 ± 0.05	0.17 ± 0.01

Data are presented as mean ± SD, *n* = 5 per group.

**Table 4:** Body weight dynamics during the 28-day treatment period, g.

Group	Sex	Day 0	Day 7	Day 14	Day 21	Day 28
Baseline	♂	30.0 ± 1.6	31.6 ± 1.6	32.8 ± 1.2	34.0 ± 1.0	34.8 ± 1.0
	♀	27.6± 1.6	28.8 ± 1.7	29.6 ± 1.6	30.3 ± 1.7	31.2 ± 1.6
AT 200 mg/kg	♂	30.4 ± 1.5	31.9 ± 1.4	32.9 ± 1.3	34.4 ± 0.9	35.1 ± 0.9
	♀	27.3 ± 1.5	28.5 ± 1.4	29.4 ± 1.5	30.1 ± 1.3	31.0 ± 1.5
AT 400 mg/kg	♂	30.1 ± 1.6	31.6 ± 1.3	32.8 ± 1.4	34.0 ± 0.8	34.9 ± 0.8
	♀	27.5 ± 1.4	28.7 ± 1.3	29.5 ± 1.6	30.2 ± 1.6	31.1 ± 1.7

Data are presented as mean ± SD, *n* = 10 per group (5 males and 5 females).

**Table 5:** Relative weight of organs after the 28-day treatment period, %.

Group	Sex	Heart	Liver	Kidneys	Spleen	Lungs	Thymus	Gonads
Baseline	♂	0.48 ± 0.02	4.70 ± 0.16	1.20 ± 0.06	0.40 ± 0.03	0.66 ± 0.04	0.15 ± 0.02	0.46 ± 0.04
	♀	0.47 ± 0.02	5.05 ± 0.19	1.12 ± 0.11	0.38 ± 0.03	0.66 ± 0.02	0.14 ± 0.01	0.075 ± 0.002
AT 200 mg/kg	♂	0.50 ± 0.01	4.75 ± 0.13	1.23 ± 0.03	0.41 ± 0.02	0.69 ± 0.02	0.16 ± 0.01	0.48 ± 0.03
	♀	0.45 ± 0.03	5.01 ± 0.16	1.09 ± 0.09	0.35 ± 0.03	0.62 ± 0.03	0.12 ± 0.01	0.069 ± 0.006
AT 400 mg/kg	♂	0.49 ± 0.02	4.74 ± 0.13	1.22 ± 0.06	0.40 ± 0.03	0.68 ± 0.04	0.15 ± 0.01	0.47 ± 0.03
	♀	0.046 ± 0.03	5.03 ± 0.19	1.10 ± 0.10	0.37 ± 0.02	0.65 ± 0.03	0.12 ± 0.02	0.071 ± 0.004

Data are presented as mean ± SD, *n* = 10 per group (5 males and 5 females).

## Data Availability

The original contributions presented in this study are included in this article, and further inquiries can be directed to the corresponding author.

## Abbreviations

The following abbreviations are used in this manuscript:

AT	CO_2_-extract–based phytopreparation from *Arctium tomentosum* Mill. roots
